# Application of KNN-based isometric mapping and fuzzy c-means algorithm to predict short-term rockburst risk in deep underground projects

**DOI:** 10.3389/fpubh.2022.1023890

**Published:** 2022-10-20

**Authors:** Muhammad Kamran, Barkat Ullah, Mahmood Ahmad, Mohanad Muayad Sabri Sabri

**Affiliations:** ^1^Department of Mining Engineering, Institute Technology of Bandung, Bandung, Indonesia; ^2^School of Resources and Safety Engineering, Central South University, Changsha, China; ^3^Department of Civil Engineering, University of Engineering and Technology Peshawar (Bannu Campus), Bannu, Pakistan; ^4^Peter the Great St. Petersburg Polytechnic University, St. Petersburg, Russia

**Keywords:** rockburst, safety, KNN, FCM, ISOMAP algorithm

## Abstract

The rockburst phenomenon is the major source of the high number of casualties and fatalities during the construction of deep underground projects. Rockburst poses a severe hazard to the safety of employees and equipment in subsurface mining operations. It is a hot topic in recent years to examine and overcome rockburst risks for the safe installation of deep urban engineering designs. Therefore, for a cost-effective and safe underground environment, it is crucial to determine and predict rockburst intensity prior to its occurrence. A novel model is presented in this study that combines unsupervised and supervised machine learning approaches in order to predict rockburst risk. The database for this study was built using authentic microseismic monitoring occurrences from the Jinping-II hydropower project in China, which consists of 93 short-term rockburst occurrences with six influential features. The prediction process was succeeded in three steps. Firstly, the original rockburst database's magnification was reduced using a state-of-the-art method called isometric mapping (ISOMAP) algorithm. Secondly, the dataset acquired from ISOMAP was categorized using the fuzzy c-means algorithm (FCM) to reduce the minor spectral heterogeneity impact in homogenous areas. Thirdly, K-Nearest neighbor (KNN) was employed to anticipate different levels of short-term rockburst datasets. The KNN's classification performance was examined using several performance metrics. The proposed model correctly classified about 96% of the rockbursts events in the testing datasets. Hence, the suggested model is a realistic and effective tool for evaluating rockburst intensity. Therefore, the proposed model can be employed to forecast the rockburst risk in the early stages of underground projects that will help to minimize casualties from rockburst.

## Introduction

Rockburst is a dynamic phenomenon which occurs in underground excavations when immense amounts of energy are released, rocks are inelastically deformed, and rocks are thrown into the excavations (1). As defined by the Mine Safety and Health Administration (MSHA), “a rockburst occurs when overstressed rock collapses abruptly, releasing large amounts of energy instantly” (2). Rockburst occurrence is mainly associated with the geological structure, properties of surrounding rock masses and lithology. It has been demonstrated that rockburst poses a severe hazard to the safety of employees and equipment in underground constructions (3–6). Therefore, for a cost-effective and safe deep underground construction or mining in burst-prone conditions, it is crucial to determine and predict rockburst intensity prioir to its occurance.

The rockburst intensity is characterized into four different levels (7). It is a big challenge to predict rockburst due to its complex and nonlinear nature. In the last few decades, several methods have been utilized to assess rockbursts (6). Monitoring and forecasting the rockburst danger are carried out by utilizing microgravity, microseismic, and geological radar techniques (8). Based on on-site monitoring of microseismic waves emitted during rock fractures, some precursory features of rockbursts were discovered that could be used to predict the risk of rockbursts. The most commonly used microseismic features to predict rockbursts are the number of events (9), energy features (10), apparent volume (11) and *b* value, which is the slope of the commutative hit toward amplitude (12). In addition to these indexes, researchers have proposed other strategies for predicting the long-term occurrence of rockburst. Rock burst risk can be assessed using tangential stress criterion (13), rock brittleness coefficient (14), strain energy storage index (15), and elastic strain energy density (16). A burst potential index based on energy has been established to assess burst proneness (17). The increasing demand for energy and construction has resulted in underground excavations being extended to greater lengths, which has caused severe rock burst disasters. It has therefore been a hot topic in recent years to examine and overcome rockburst risks.

In the previous few decades, rockburst prediction or evaluation approaches have evolved, but there has never been a breakthrough or generally recognized method that has been preferred over others. Since then, the rockburst has been an unsolved and alarming issue that needs to be resolved more precisely. In order to eliminate the threat of rockbursts in the first place, advanced rockburst prediction is crucial to reducing the cost of the damage and preventing major losses from a rockburst. In recent years, state-of-art intelligent techniques have widely been implemented to overcome the severe dynamics hazards of rockburst disasters for the safe installation of underground projects. The researchers have extended their horizons and utilized cutting-edge soft-computing methods to predict the rockburst occurrence intensity successfully. Additionally, the intelligent algorithm is low cost, only focuses on input and output parameters, and has broader applicability (18–21).

The development of artificial intelligence makes the intelligent system more suitable for rockburst prediction. Based on distinct geological conditions, different models are proposed, and these models are so specialized that they cannot be used concurrently to many projects. Rockburst is affected by non-linear factors, and artificial intelligence algorithms are outclassed in non-linear analysis with high-dimensional datasets. The most widely used methods to predict rockbursts are support vector machines (SVMs), artificial neural networks (ANNs), K-nearest neighbors (KNNs), classification and regression trees (CARTs), ensemble learning and random forests (RFs) (22). The results of a study have shown that ANN models can be used to predict rockburst risks in deep gold mines in South Africa after an improved model was introduced (23). Zhao et al. (24) constructed a data-driven model using a convolutional neural network (CNN) and compared its performance with that of a traditional neural network. An SVM model was used by Zhou et al. (22) to categorize a long-term rockburst. The four classical single intelligent algorithms, namely k-nearest neighbors (KNN), SVM, deep neural networks (DNN) and recurrent neural networks (RNN), were combined to form four ensemble models (KNN–RNN, SVM–RNN, DNN–RNN and KNN–SVM–DNN–RNN) using stacking ensemble learning (25). A new probability model for tunnel rock burst prediction was proposed based on Copula theory and the least square support vector machine (LSSVM) method optimized *via* particle swarm optimization (PSO) (26). Using kernel principal component analysis (KPCA), adaptive-PSO, and SVM, Li et al. (27) developed a hybrid model (KPCA-APSO-SVM). The short-term rockburst risk was predicted using t-distributed stochastic neighbor embedding (t-SNE), K-means clustering, and extreme gradient boosting (XGBoost) algorithms (28). On the basis of microseismic monitoring data, Zhao et al. (29) developed a model for prediction of rockbursts that uses a decision tree (DT) model. For assessing the rockburst hazard of an active hard coal mine, Wojtecki et al. (1) used neural networks, decision trees, RF, gradient boosting, and XGBoost. In order to study the predictability of short-term rockburst, Liang et al. (30) used microseismic data from Jinping-II hydropower project to examine the predictability of short-term rockburst. This study evaluated several ensembles learning algorithms, including RF, adaptive boosting, gradient boosting decision tree, XGBoost, and light gradient boosting machine (LightGBM). RF and GBDT have shown good performance. Li et al. (6) demonstrated the predictability of different ensemble trees in estimating rockburst based on 314 real rockbursts. Sun et al. (31) proposed an RF and firefly algorithm (FA) based ensemble classifier to achieve an optimum rockburst prediction model. A study by Ahmad et al. (32) investigated that J48 and random tree algorithms can successfully predict the rockburst classification ranks based on 165 rockburst cases.

In deep underground projects, a self-organizing map and fuzzy c-mean clustering techniques were used to cluster rockbursts events (33). Even though several rockburst estimation models have been described and compared by previous researchers (34–40), developing an accurate and reliable predictive model still poses a significant challenge for the ground, which is likely to experience frequent rock bursts. Further, many other models for forecasting rockbursts can be considered valuable and efficient tools for geological and mining engineering applications. Unsupervised machine learning is an approach that researchers have shown increasing interest in adopting, in which data labels are not required to be known in advance to perform the analysis. In order to classify the data, clusters are formed based on their proximity to each other and the distance from each cluster's center is taken into account (28, 29, 41). So far, the available studies have succeeded in predicting and classifying the rockburst dynamic disaster but were never entirely successful. A particular procedure can be appropriate in certain instances but not in others. Therefore, the adaptation of state-of-art data depletion combined with unsupervised and KNN learning has less contribution to rockburst intensity prediction. Table 1 illustrates the summary of previously published literature to predict rockburst.

**Table 1:** Summary of previously published literature to predict rockburst.

**Year**	**Number of input features**	**Number of datasets**	**Approaches**	**Accuracy (%)**	**References**
2020	6	93	XGboost	73.33	(30)
2020	6	93	Gradient boost decision tree	76.67	(30)
2020	6	93	Adaboost	66.67	(30)
2020	6	93	Random forest	80	(30)
2021	4	165	J48	92.857	(32)
2018	3	108 and 132	Decision tree model	73-93	(42)
2008	4	36	AdaBoost	87.8-89.9	(43)
2008	6	45	v-support vector regression	93.75	(44)
2012	6	132	Heuristic algorithms and support vector machine	66.67-90	(22)
2021	6	311	Scorecard methodology	75	(45)

## Significance of the study

The predicting features of rockburst levels vary throughout a wide range of rock engineering, mining and geotechnical engineering projects. The impacts of each uncertainty level are yet unclear. In fact, many different findings are reported in the diverse domains of rockburst prediction, short-term rockburst is a complex and sophisticated phenomenon that cannot yet be accurately predicted.

**Figure 1:**
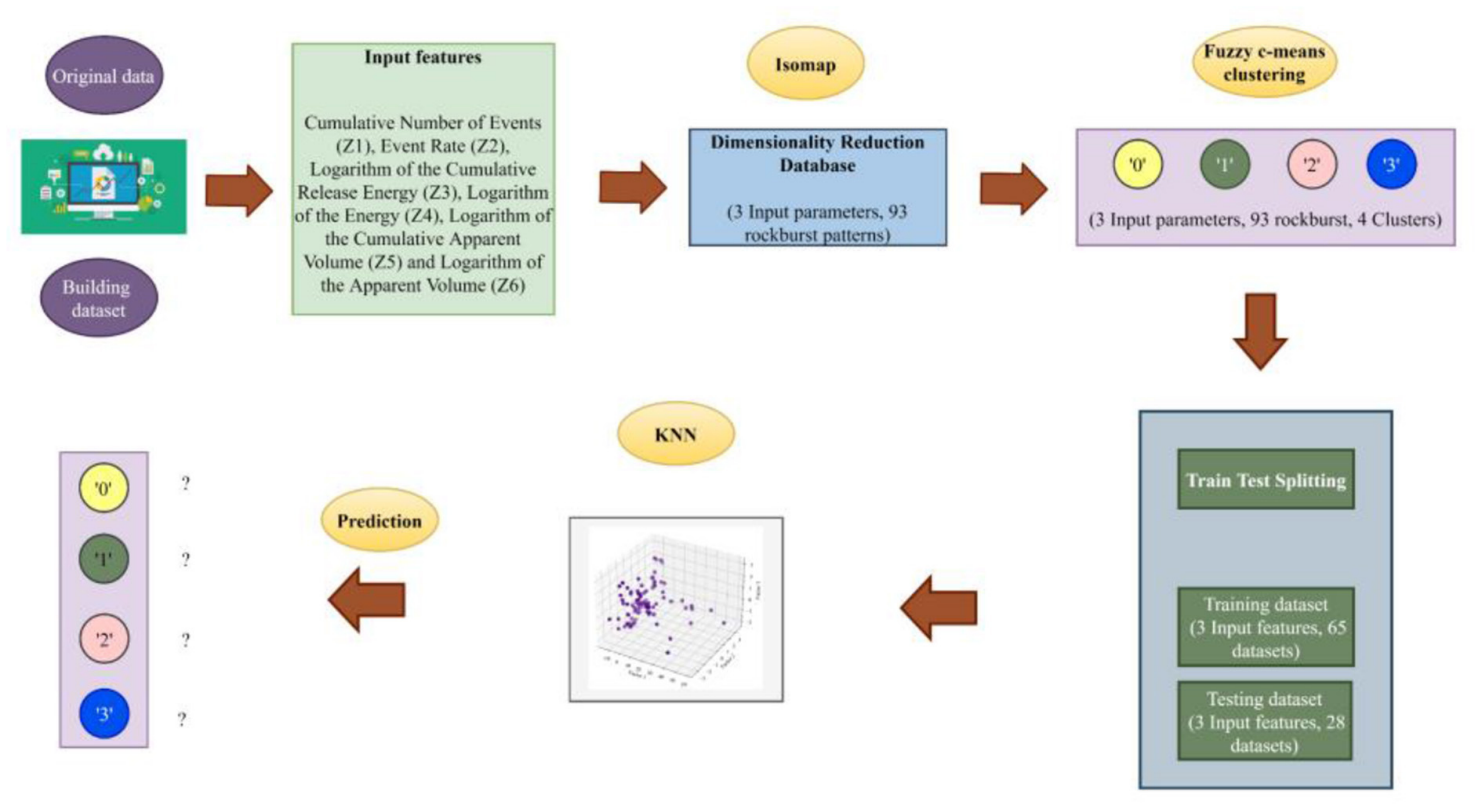
Flowchart of the proposed study.

This study develops short-term rockburst prediction model based on uncertainty incorporating unsupervised and supervised learning in order to apply the model effectively in addressing the rock engineering problems. The following three steps are provided in this study to forecast the short-term rockburst:

1) To begin with, the original rockburst database's magnification was reduced using a state-of-the-art method called isometric mapping.
2) The isometric mapping dataset was categorized using fuzzy c-means clustering as an unsupervised machine learning approach to reduce the minor spectral heterogeneity impact in homogenous areas.
3) In order to anticipate different levels of short-term rockburst datasets, KNN, a supervised machine learning has been designed. The study's flowchart is shown in Figure 1.

## Materials and methods

### Data curation

**Figure 2:**
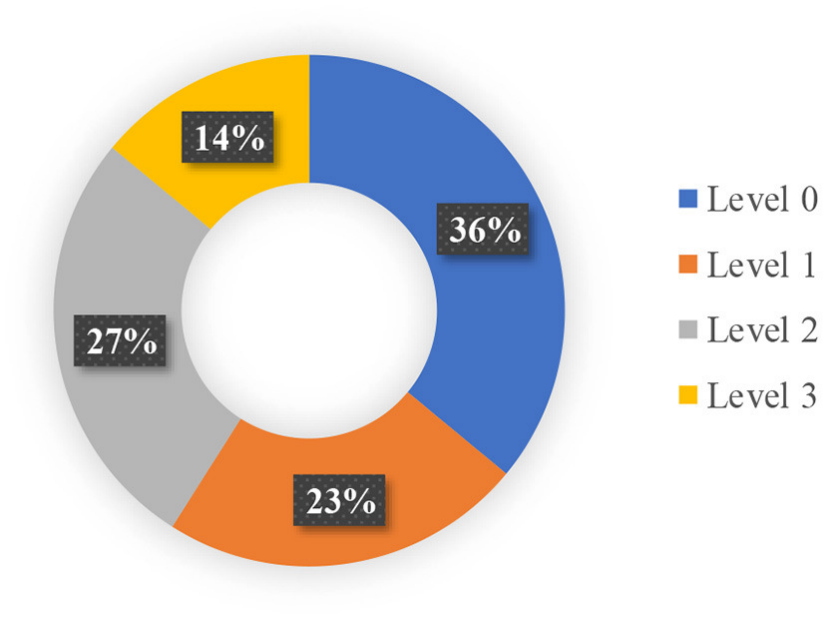
The distribution of different rockburst level.

The database for this study was built using authentic microseismic monitoring occurrences from the Jinping-II hydropower project in China. The Jinping II Hydropower Station is located close to the boundaries of Muli, Yanyuan, and Mianning in the Liangshan Yi Autonomous Region of Sichuan, southwest China. It is an ultra-deep buried long tunnel with an extra-large subterranean water power engineering. The average length of the cave line is around 1,667 km, the width of the excavation hole is 13 m, the underlying rock mass is normally buried between 1,500 and 2,000 m, and the maximum buried depth is roughly 2,525 m. The excavation tunnel section of the 1# and 3# diversion tunnels is a four-hearted horseshoe with an excavation diameter of 13 m, whereas the tunnel boring machine (TBM) excavation section of the 1# and 3# diversion tunnels is a circular section with an excavation diameter of 12.4 m. The distance between the four diversion tunnels is 60 m. The auxiliary tunnels A and B are 35 m apart from the centerline of the construction drainage tunnel, whereas the construction drainage tunnel is 45 m apart from the 4# diversion tunnel (26). The database consists of 93 short-term rockburst occurrences with six influential features (46). The dataset analyzed in this study was obtained from the work of Liang et al. (30) and was built on the dataset readily accessible by Feng et al. (46). Rockburst intensity has been divided into four categories: no rockburst level (represented by Level 0) illustrates that the rock composites have no considerable breakage on the free face; slight rockburst level (represented by Level 1) indicates minor composites with modest fragment movement and kinetic energy transfer; moderate rockburst level (represented by Level 2) illustrates the sample debonding of the rock mass inside the diverticulum and highway structure; while severe rockbursts level (represented by Level 3) involve a significant amount of rock mass cracking that immediately fractures the nearby rock mass. The distribution of different rockburst level in this study is shown in Figure 2. Table 2 depicts the statistic of input and output features employed in the rockburst database.

**Table 2:** The statistic of input and output feature in rockburst database.

**Rockburst level**	**Cumulative number of events Z1 (Unit)**	**Event rate Z2 (unit/day)**	**Logarithm of the cumulative release energy Z3 (J)**	**Logarithm of the energy Z4 (J/day)**	**Logarithm of the cumulative apparent volume Z5 (m3)**	**Logarithm of the apparent volume Z6 (m3/day)**
3	41	3.727	4.694	3.653	4.926	5.968
2	14	1.556	4.622	3.668	4.887	5.841
2	17	1.889	4.397	3.443	3.8	4.754
2	18	1.8	4.703	3.703	4.295	5.295
…..	…..	…..	…..	…..	…..	…..
2	36	2.571	4.336	3.16	2.583	4.729
1	8	2.667	3.977	3.5	4.727	5.204
1	16	2.667	4.681	3.903	2.843	3.621
0	6	1.5	2.735	2.133	4.698	5.3
Minimum	1	0.11	0.78	0.178	2.511	1.66
Maximum	70	12.25	7.094	5.89	5.168	4.39
Mean	13.011	1.735	4.389	3.562	4.15	3.334
Standard deviation	13.69	1.738	1.441	1.332	0.66	0.558

### Data visualization

**Figure 3:**
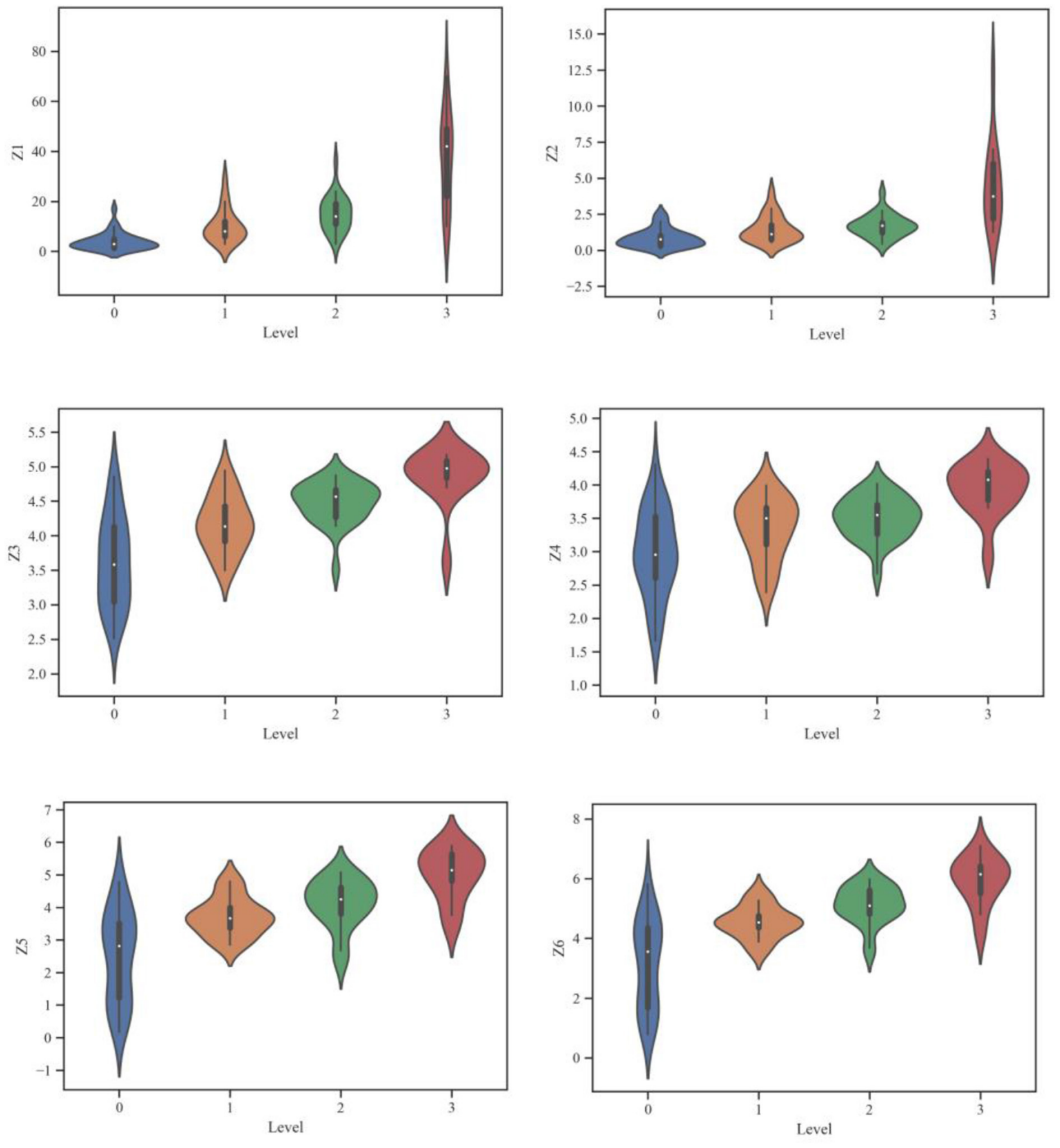
Voilen chart of significant features employed in the study.

Table 2 reveals that this study incorporates six significant features. The values of Z1, Z2, Z3, Z4, Z5 and Z6 are adjusted on a logarithmic scale to make the effective implementation more favorable. The log parameter's primary objective is to address the database's skewness toward big data. The rockburst database has been investigated by utilizing Python programming language. The Python programming language offers important assistance for experimental data mining, together with data input classification and statistical learning techniques evaluation. Additionally, the learning outcome for a big amount of data is made apparent by visualizing the input data (47). Figure 3 depicts the voilen of several features for the four rockburst levels. Figure 3 shows a positive correlation between each feature and the corresponding rockburst level. The higher level of rockburst is indicated by the larger values of the features. Additionally, a few outliers may be seen in all of the short-term rockburst dataset's features for accompanying rockburst intensity, demonstrating the heterogeneity of the rockburst events. Therefore, this study incorporates the effects of all the features to increase the authenticity of the rockburst data structure.

### Isometric mapping (ISOMAP)

**Figure 4:**
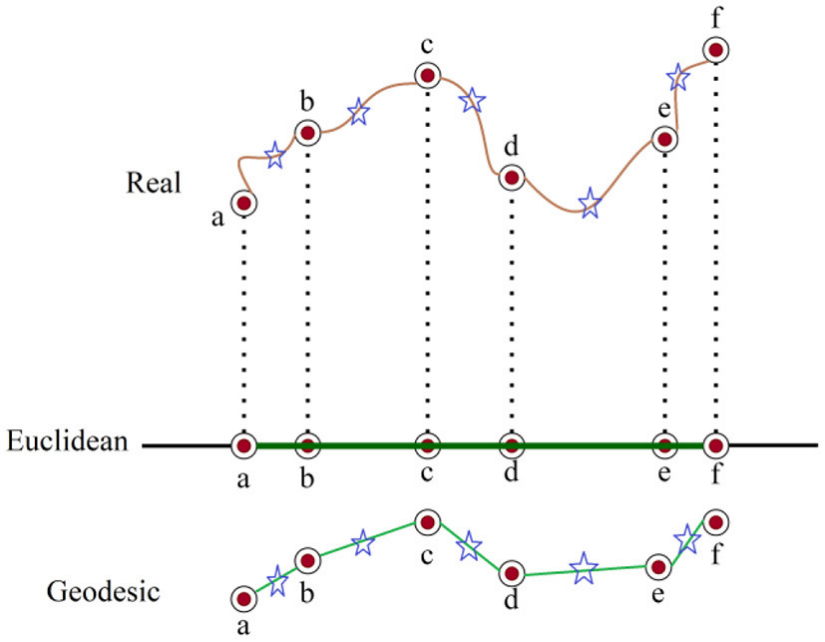
The mechanism of Isomap.

Isometric Mapping (ISOMAP) is a stochastic technique for reducing dimensionality that maintains geodesic adjacency using a non-Euclidean measure. As a result, it preserves nonlinear characteristics of the original data that are lost in conventional analysis (48). Although the ISOMAP represents nonlinear fluctuations in the broader domain, it preserves linearity in small domains (49). Alternatively, this is referred to as multifaceted learning or consideration. It follows that the manifold's small local area is a conversation of metric space (50). Figure 4 depicts the mechanism of ISOMAP. The relevant datasets are denoted by the letters *a–f*. They have star points as neighbors. The measured distances of these data are represented by the green segments. It works by mapping the original dataset into a predetermined low-dimensional embedded space and makes the assumption that the high dimensional data is uniformly sampled from a uniform manifold. This method then attempts to identify the underlying manifold (51).

The standard Euclidean geometry and domain-specific metrics can both be used to approximate the geodesic distance. A series of brief hops between nearby points can be added up to approximate domain-specific distance, and the input space's standard Euclidean metric gives a decent approximation of geodesic distance (52). With the ability to learn a wide range of nonlinear manifolds, ISOMAP combines the key algorithmic characteristics for computational effectiveness, global optimality, and guarantees the asymptotic convergence (53).

### Fuzzy c-means algorithm (FCM)

**Figure 5:**
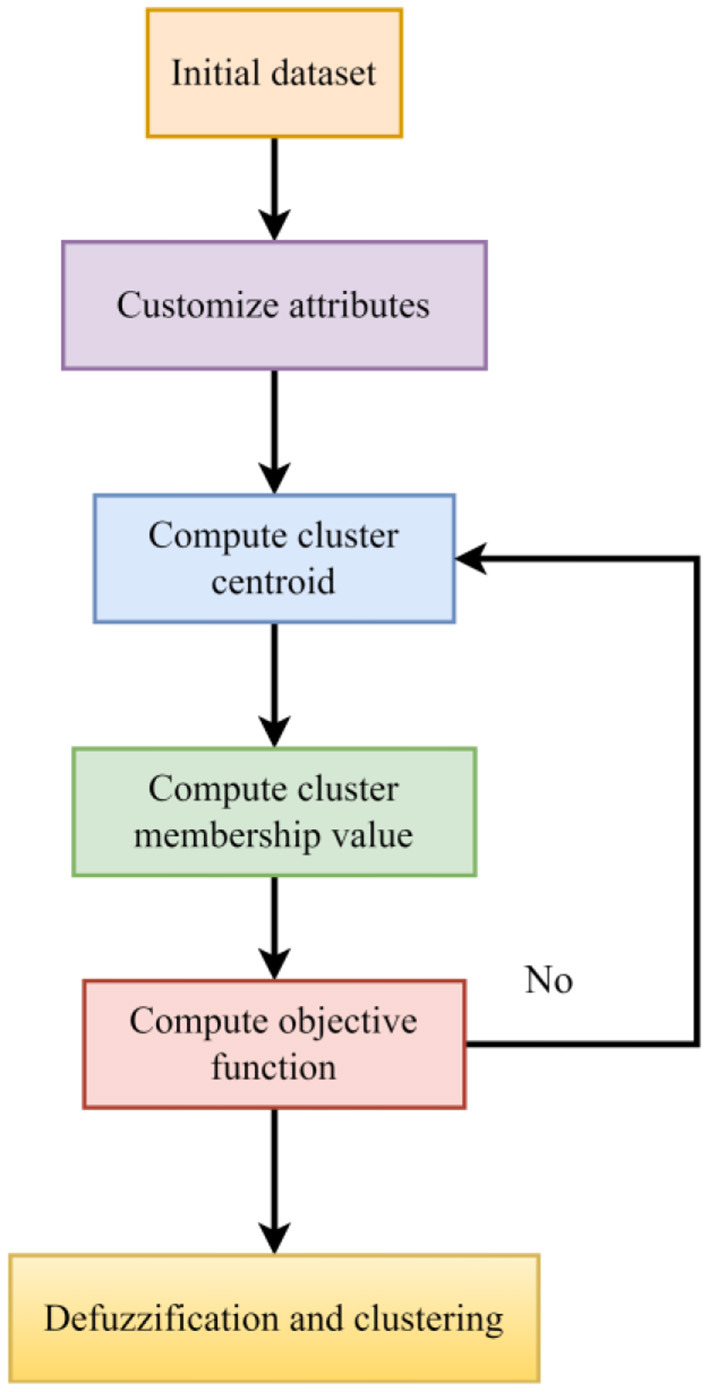
Flowchart of fuzzy c-means algorithm.

The theory of the fuzzy set was developed for the purpose of precisely resolve the difficulties of certainty and uncertainty in the field of optimization and artificial intelligence (54, 55). The FCM was developed based on clustering analysis concept which allows every level to classify in many categories knows as fuzzy sets. This algorithm often has significant advantages over more conventional methods. Several researchers have proposed innovative strategies to FCM in order to solve the problems related to different fields (56–59). Figure 5 depicts the flowchart of FCM algorithm.

### K-nearest neighbor (KNN)

The K-Nearest neighbor (KNN) algorithm is a supervised technique that is often utilized in a wide range of situations due to its efficiency and simplicity. The most modern development highlights KNN's potential for reducing distortion in a dataset (60–63). Big data analysis with a KNN classifier demands powerful computing resources. According to the classification approach, a test sample's class label is established using the *k* nearby samples from the training dataset (64). The connection between all training instances and the testing data ought to be determined in order to perceive the *k* nearest neighbors. Each test instance is allocated using KNN contingent on its *k* closest neighbors. The separation between all training instances and the test instances ought to be computed in order to find the *k* nearest neighbors (63).

Equation 1 is employed in the KNN to determine the spectral intervals (Euclidean distances) across each unknown value and the samples plots. The *k* sample plots that are closest to the estimated level are chosen based on the aforementioned particular node.


$$\gamma =\sqrt{\sum_{k=1}^{m}{\left(u_{k}-v_{k}\right)}^{2}}\text{ Eqn}.\;\text{1}$$

whereas γ stands for the spectral distance between the *u* and *v* in the n-dimensional space, *u*_*k*_ and and *v*_*k*_ are the spectral values of unknown variables and *v* in the *kth* selected spectral variable, respectively. By weighing the reciprocals of their spectral distances from the ideal *k* closest plots, the rockburst level was predicted using Equation (2).


$$\begin{array}{l} Level_{p}=\;\frac{\sum_{z=1}^{y}\frac{1}{d_{\gamma z}}*v_{z}}{\sum_{z=1}^{y}\frac{1}{d_{pz}}}\text{ Eqn. 2} \end{array}$$

whereas *Level*_*p*_ represents the appropriate forecasted level at the *p*, *v*_*z*_ depicts the rockburst observation associated with to the *zth* sample, *d*_*pz*_ represent the spectral interval from the *P* to the *zth* samples, and *k* illustrates the ideal number of samples.

**Figure 6:**
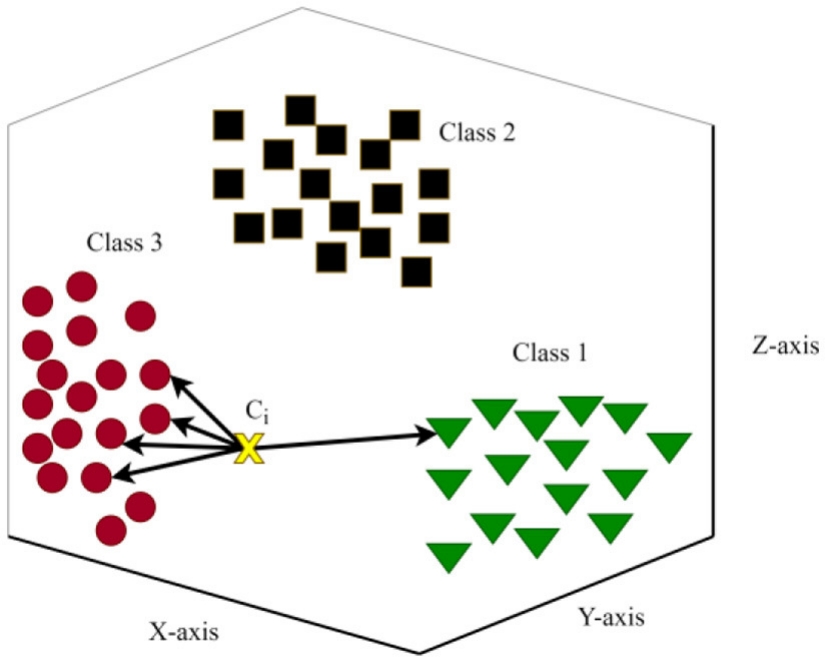
A simple 3D multi-class KNN model for the rockburst dataset.

Finally, the suggested KNN algorithm with the rockburst data has been computed on Algorithm 3. The massive training data is divided into *m* distinct sections first. The size and dispersion of each cluster throughout the various axes of the data space ($d_{u}^{t}$) should be estimated once the clusters have been identified. The most appropriate cluster of rockburst dataset may be chosen using Algorithm 1. Selecting the appropriate data cluster can substantially affect the outcome of the classification process because the KNN process' output is dependent on the training data. In order to determine the *k* nearest neighbors and determine the rockburst level of the test sample, the KNN algorithm is applied to the chosen portion of the training data (65). A simple 3D multi-class KNN model for the rockburst dataset is shown in Figure 6.

## Result and discussion

Large-scale theoretical and mathematical analyses of datasets are performed using the powerful programming language Python. The clustering and classifying focused data mining techniques and algorithms are supported by the Python programming language. This option is one of the best platforms for designing scalable applications since it has so many advantageous characteristics. Therefore, it may be used to the framework of big data analysis in large rockburst datasets to produce reliable findings.

**Figure 7:**
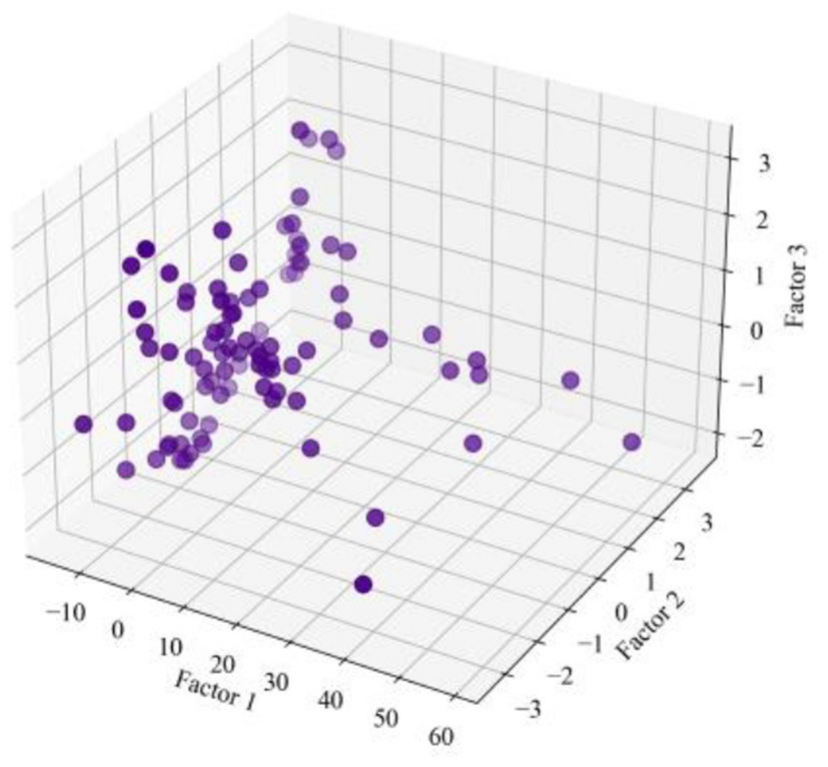
3D visualization of ISOMAP based rockburst database.

The ISOMAP technique has been implemented as a method for learning a nonlinear manifold from a collection of unstructured high-dimensional datasets. Its foundation is an expansion of the conventional multidimensional scaling approach to data reduction. The ISOMAP enhances the data identification and make it simple to quantify differences between data points and the reconstructed space. In comparison to the distances in the other conventional data dimensionality reduction rebuilt spaces, the separation far or near in the ISOMAP-reconstructed matrix give the dimension of the similarity intervening the data point. The ISOMAP effectively separates the points by calculating the geodesic distance. Several ISOMAP iterations can assist in separating the data points, allowing them to be grouped into various clusters. The benefit of ISOMAP is that it employs the Dijkstra's algorithm, which determine the optimum route through neighbors and terminate at every point, to provide realistic distance estimates between the interconnected points distance, which accurately depicts the change in actual space distance between the data points. ISOMAP tool is used to visualize the original rockburst database from high-resolution matrix to low-resolution matrix. The original rockburst dataset having six influential feature was employed in this study. The proposed ISOMAP technique is implemented in Jupyter notebooks. The ISOMAP's three leading factors were utilized to depict the rockburst points that had been reduced after dimensionality reduction. The original rockburst data points in the ISOMAP reconstructed 3D structure showed greater spatial variation, which were evenly spaced apart. Table 3 represent three influential factors rockburst acquired from ISOMAP. The 3D structure of the isometric reconstructed rockburst dataset is shown in Figure 7.

**Table 3:** The influential factor of rockburst dataset acquired from isomap.

**Pattern no**.	**Factor 1**	**Factor 2**	**Factor 3**
1	29.63107	1.098796	−0.24922
2	1.649354	−1.04534	1.085205
3	4.826978	−0.45574	0.016694
4	5.97017	−0.5952	−0.01854
5	−2.48045	−0.55883	0.118498
…..	…..	…..	…..
89	17.77873	−1.29822	−0.75349
90	24.13729	1.433746	0.084803
91	−4.68471	−1.88809	2.039045
92	3.84182	1.570014	1.10249
93	−7.98256	−2.45747	2.358197

The application of FCM could be used to solve a wide range of geostatistical data analysis issues. Each type of numerical data can be employed to generate prototypes and fuzzy partitions with this programmed. These partitions are helpful for supporting established substructures or pointing to substructure in undiscovered data. The FCM clustering with ISOMAP reconstructed points has been employed to determine whether it is feasible to categorize the rockburst level. The FCM clustering is implemented in Jupyter notebook. Researchers have established the cluster monitoring's generalizability of the findings (29). A metric used to determine the effectiveness of a clustering method is the silhouette coefficient, often known as the silhouette score (66, 67). Its value is between −1 and 1. The score 1 clusters are clearly distinguishable and spaced far apart, score 0 indicates that clusters are undifferentiated or that there is no statistically significant difference across clusters, whereas score −1 implies that the clusters are assigned incorrectly. Equation 3 depicts the silhouette score.


$$\begin{array}{l} Sillhouette\;score\;=\;\frac{\left(y-x\right)}{\max\left(y,x\right)}\text{ Eqn}.\;3 \end{array}$$

Whereas *y* is the average distance between all clusters and *x* is the average intra-cluster distance, or the average distance between each point inside a cluster.

**Figure 8:**
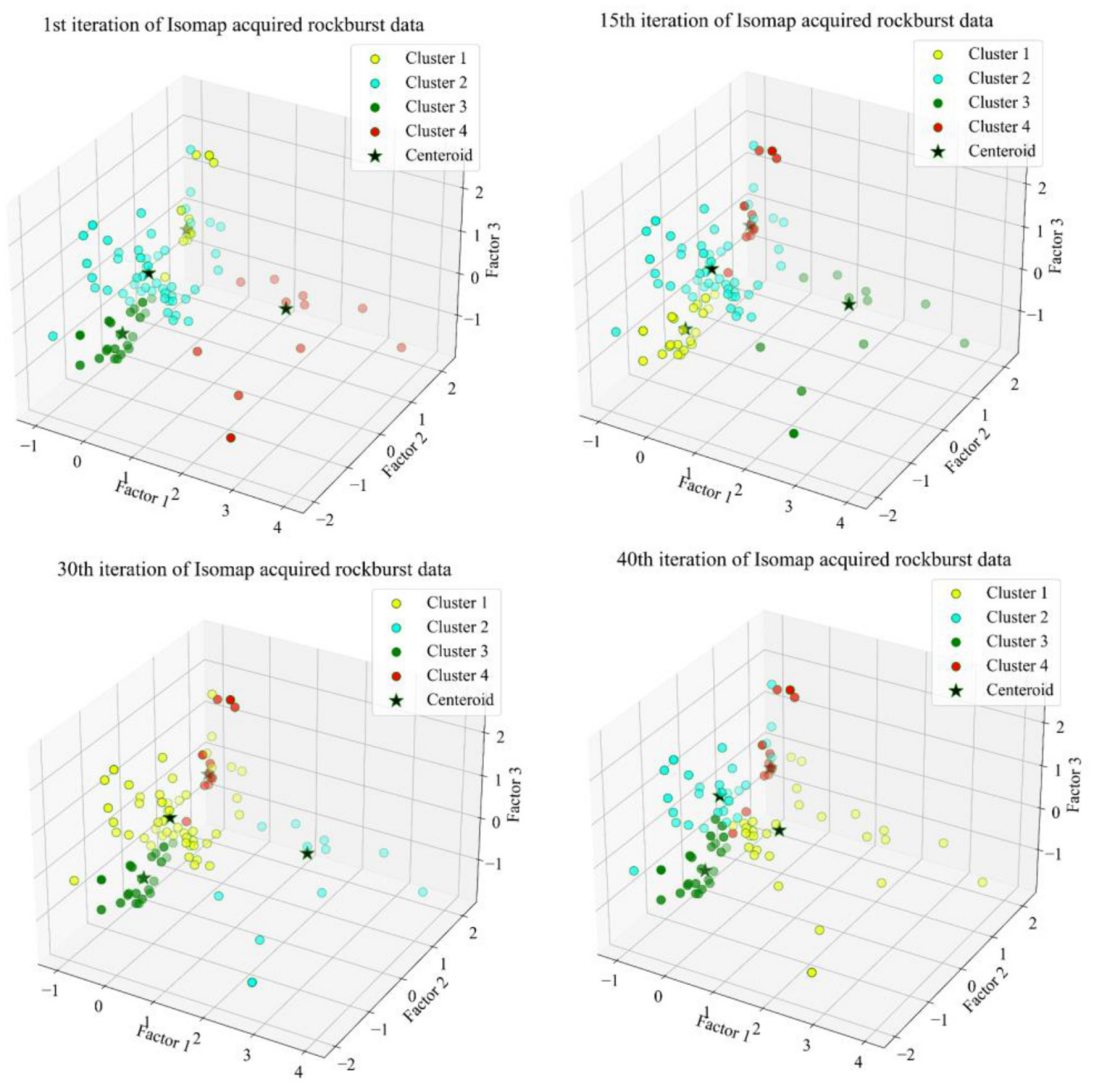
Fuzzy c-means algorithm visualization on Isomap database.

The silhouette score can demonstrate that the ISOMAP acquired data is correctly categorized, representing the arrangement of the features into the categories to which they belong. This index measures the effectiveness of the clustering's authentication in choosing the best *k* cluster members. We suppose that there will be four FCM clusters, which corresponds to the four distinct rockburst intensities. This study calculated many iterations phases, as illustrated in Figure 8. The yellow, aqua, green and red color were selected to identify the rockburst level 0, 1, 2 and 3 respectively. A suitable model for clustering has been demonstrated in several studies to have a silhouette score of higher than 0.5 (68–70). Following the tenth iteration in the Isometric mapping derived short-term rockburst dataset, the silhouette score of 0.53 demonstrates that the clusters were consistent and authentic. The center of the circle of the four clusters were (0.740, −0.08, 0.035), (−0.139, −0.543, 0.864), (−0.581, −0.419, −1.136), (−0.776755, 1.755, −0.212). Based on the performance evaluation, the FCM performed well to categorize four different levels of rockburst.

The KNN is a supervised machine learning method that is appropriate for classification and modeling and is reasonably simple to construct. It may be employed in a variety of rock engineering applications. It selects *k* sample plots from the training dataset that are most similar to the predicted rockburst level, and it then utilizes the “feature similarity” concept to weight the observations of the *k* plots based on how similar their significance is to the unknown rockburst level. The KNN technique for predicting short-term rockburst levels was implemented using the Python programming language. Each unknown rockburst level in this study was given a Euclidean distance to each sample plot, and these distances were used to determine the spectral distances.

The FCM acquired data were randomly divided into training datasets (70%) and testing datasets (30%). The training dataset is used to develop and verify the framework while the testing datasets are utilized in order to assess the framework's capability to estimate the rockburst levels that use previously unobserved data. On the testing dataset, the KNN's forecasting outcomes were obtained. Various performance indices including precision, recall, and F1-score have been employed by the researchers to evaluate the performance of a classification model (71). In this study, precision, recall, and F1-score have been used to predict the outcomes of the proposed KNN algorithm when associated with ISOMAP and FCM.

The Python programming language was used to generate the classification report for the testing dataset. The classification report provides insight into the effectiveness of the framework on the rockburst events, which is depicted in Table 4. The testing dataset had almost the accuracy of 96% confirming the approximate fitting of the model.

**Table 4:** The classification report of the proposed approach on the rockburst database.

	**Precision (%)**	**Recall (%)**	**F1-score (%)**
Level 0	100	92	96
Level 1	86	100	92
Level 2	100	100	100
Level 3	100	100	100
Accuracy			96
Macro avg	96	98	97
Weighted avg	97	96	96

In comparison to level 1, the precision value for level 0, 2 and 3 produced superior results. In terms of precision, the level 0, 1, 2, and 3 have values of 100, 86, 100, and 100%, respectively. The recall values for levels 1, 2, and 3 outperformed level 0 in terms of findings. In comparison to level 0 and 1, the F1-score value for level 2 and 3 attained superior results. The average and weighted recall scores could reach high values of 98 and 96% as a trade-off between precision, recall, and F1-score. The accuracy of the testing dataset was 96%, supporting the proposed model's approximation of fitting. Hence, the proposed KNN based ISOMAP and FCM algorithm demonstrates favorable classification results for the developed KNN model in successfully identifying the rockburst risk in underground civil structures.

**Figure 9:**
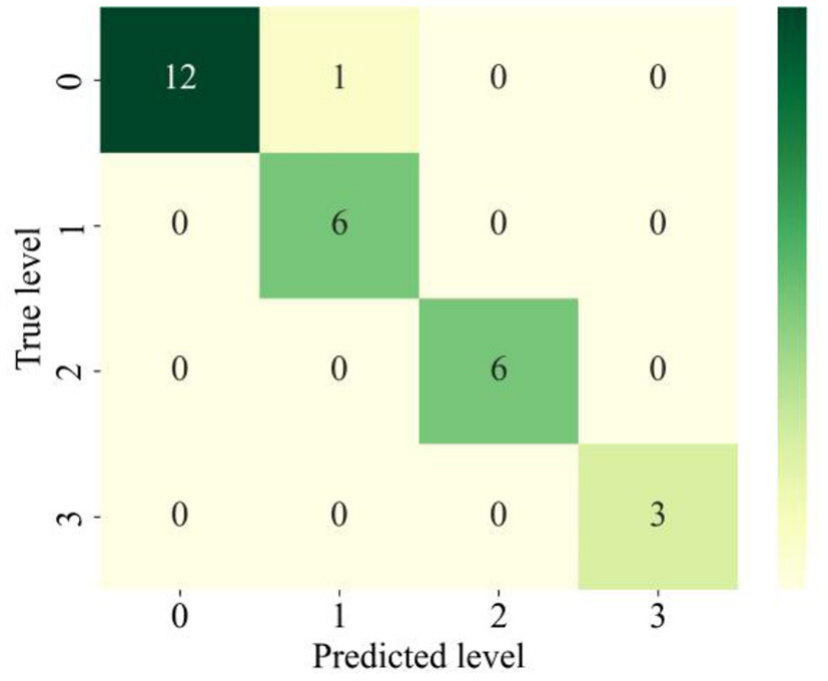
Confusion matrix of KNN on testing dataset.

Figure 9 demonstrates the confusion matrix that's been generated for the KNN algorithm. The data on the primary diagonal represent the number of samples that the KNN accurately predicted. As can be observed, the KNN successfully classified the majority of rockburst samples. In the whole short-term rockburst dataset, only one rockburst level has been incorrectly predicted. In more detail, one level (0) is incorrectly labeled as level (1). Hence, the KNN algorithm performed well in forecasting the rockburst level in underground civil structures.

## Conclusion

This study developed ISOMAP + FCM + KNN framework to effectively and accurately anticipate rockburst levels. By examining the results for the proposed model using several performance metrics, the robustness of the generated framework was demonstrated. During this study, three approaches that are frequently used in geotechnical engineering—ISOMAP, FCM, and KNN model—were used to forecast the rockburst level. More specifically, the data used in this study is collected from several microseismic monitoring occurrences. The statistical performance is used to assess the short-term rockburst level in order to approximation the resilient framework for the best effective model in connection with data prediction. The results of the ISOMAP, FCM, and KNN model demonstrate that it is capable of generating highly precise predictions of the rockburst level.

As a result, it is recommended to employ the ISOMAP + FCM + KNN model developed in this study as a reliable and effective model for predicting the intensity levels of rockbursts. Due to the suggested model's accurate prediction performance in various rock environments, it can be used as a rockburst mitigation and warning system. By keeping several additional geological and rock mechanics data, the model can be made more comprehensive. This model can be utilized as a practical and useful tool for determining rockburst risk. The suggested model can be used to predict the level of rockburst in the early stages of underground mining projects in order to reduce the injuries and fatalities from rockburst.

It is important to take into account the range and volume of trainings because this has an impact on the data-driven models' ability to make logical inferences. The proposed model will be further expanded by developing certain cutting-edge machine learning techniques and contrasting the results of those models with the results of the model attained in this work.

## Data availability statement

The original contributions presented in the study are included in the article/supplementary material, further inquiries can be directed to the corresponding author.

## Author contributions

MK: conceptualization, data curation, software, and visualization. MK and BU: writing—original draft. MK, BU, MA, and MS: validation and formal analysis. MA and MS: supervision and project administration. MS: funding acquisition. All authors have read and agreed to the published version of the manuscript.

## Funding

This research is partially funded by the Ministry of Science and Higher Education of the Russian Federation under the strategic academic leadership program Priority 2030 (Agreement 075-15-2021-1333 dated 09/30/2021).

## Conflict of interest

The authors declare that the research was conducted in the absence of any commercial or financial relationships that could be construed as a potential conflict of interest.

## Publisher's note

All claims expressed in this article are solely those of the authors and do not necessarily represent those of their affiliated organizations, or those of the publisher, the editors and the reviewers. Any product that may be evaluated in this article, or claim that may be made by its manufacturer, is not guaranteed or endorsed by the publisher.
